# Identification of the NLS and NES motifs of VP2 from chicken anemia virus and the interaction of VP2 with mini-chromosome maintenance protein 3

**DOI:** 10.1186/1746-6148-8-15

**Published:** 2012-02-07

**Authors:** Jai-Hong Cheng, Shyang-Chwen Sheu, Yi-Yang Lien, Meng-Shiunn Lee, His-Jien Chen, Wen-Hong Su, Meng-Shiou Lee

**Affiliations:** 1 Department of Medical Research, Chang Gung Memorial Hospital-Kaohsiung Medical Center, Chang Gung University College of Medicine, Kaohsiung, Taiwan; 2 Department of Food Science, National Pingtung University of Science and Technology, Pingtung, Taiwan; 3 Department of Veterinary Medicine, National Pingtung University of Science and Technology, Pingtung, Taiwan; 4 Department of Medical Research, Tung's Taichung Metro Harbor Hospital, Taichung, Taiwan; 5 Department of Safety, Health and Environmental Engineering, Mingchi University of Technology, Taipei, Taiwan; 6 School of Chinese Pharmaceutical Science and Chinese Medicine Resources, China Medical University, Taichung, Taiwan

## Abstract

**Background:**

VP2 of chicken anemia virus (CAV) is a dual-specificity phosphatase required for virus infection, assembly and replication. The functions of the nuclear localization signal (NLS) and nuclear export signal (NES) of VP2 in the cell, however, are poorly understood. Our study identified the presence of a NLS in VP2 and showed that the protein interacted significantly with mini-chromosome maintenance protein 3 (MCM3) in the cell.

**Results:**

An arginine-lysine rich NLS could be predicted by software and spanned from amino acids 133 to 138 of VP2. The critical amino acids residues between positions 136 and 138, and either residue 133 or 134 are important for nuclear import in mammalian cells based on systematic mutagenesis. A NES is also predicted in VP2; however the results suggest that no functional NES is present and that this protein is CRM1 independent. It was also shown that VP2 is a chromatin binding protein and, notably, using a co-immunoprecipitation assay, it was found that VP2 association with MCM3 and that this interaction does not require DSP activity.

**Conclusions:**

VP2 contains a NLS that span from amino acids 133 to 138. VP2 is a CRM1 independent protein during nuclear export and associates with MCM3 in cells.

## Background

CAV is a small non-enveloped, single-stranded, circular DNA virus and was first isolated in Japan [1]. This virus belongs to the genus Gyrovirus of the Circoviridae family and causes a severe immunosuppressive syndrome and anemia in chickens [2]. It is a ubiquitous pathogen of chickens and has a worldwide distribution. According to epidemiological studies, it has been shown that almost all newly hatched chicks are susceptible to CAV as a clinical syndrome, but not mature chickens [3]. Normally, young chickens, generally fewer than 2 weeks of age, are very susceptible to this virus through vertical transmission via hatching eggs [4]. The virus typically induces aplasia of the bone marrow and damage to lymphoid tissue, which causes anemia and acute immunodeficiency syndrome [5,6]. Up to the present, a large number of isolates, including strains from Australia, Bangladesh, Brazil, China, Germany, Malaysia, Nigeria, Slovenia, Taiwan and USA, have been reported and have had full or partial sequences published [7-9].

The DNA genome of CAV is about 2.3 kb in size [10-12] and there are three ORFs present on the negative sense genome. At least three proteins are produced from a single polycistronic 2.1 kb mRNA that is reliable produced as a single molecule and contains a promoter, TATA-box, and poly (A) signal [11,13,14]. The three translated proteins are called VP1, VP2 and VP3. VP1 is a 51 kDa protein that is the structure protein involved in assembly of the viral caspid [15]. VP2 is a 24 kDa protein that contains a dual-specificity phosphatase (DSP) activity and some apoptotic activity [2,16,17]. However, VP2's apoptotic activity is much weaker than that of VP3. VP3 is a 13 kDa protein, also named apoptin, which induces apoptosis in infected chicken cells and human tumor cell lines [2,18,19]. During virus infection, VP2 and VP3 are detected very early, namely 12 h post infection, while VP1 is detected only after 30 h post infection [16]. Some additional proteins have been reported to be translated after further splicing of the mRNA, but their biological functions have not been elucidated [20].

As mention above, VP2 is a DSP enzyme [17]. The key catalytic residues of active site have been identified to be serine, threonine, and tyrosine. The cysteine residues, respectively, are located at positions 95 and 97 in the catalytic motif of VP2. Furthermore, mutation of these residues to serine results in reduced virus replication efficiency in the cell [17]. This effect indicates that the phosphatase activity of VP2 is required for virus replication. It has been reported that co-expression of VP1 and VP2 allows neutralizing antibodies to be raised [21,22] and it has been suggested that VP2 is a scaffold protein [23] that corrects the conformation of VP1. Therefore, it is expected that VP2 is a multifunctional protein with roles in virus infection, assembly and replication. VP2 possess a putative NLS and the protein is known to accumulate to a large extent in the nucleus of infected chicken cells [16,24]. A recent study has shown that VP2, when fused to GFP, shows nuclear localization and this result indicates that the NLS of VP2 is also functional in plants [25].

Until now, the specific mechanism for the cellular localization of VP2 is not well understood. In this study, we first use bioinformatics to analysis the amino acid sequence of various different isolates of VP2, and were able to predict and examine for the presence of putative NLS and NES motifs, which have never been characterized previously. We generated GFP fused to various versions of VP2 created by truncation, site directed mutagenesis, and multiple site directed mutagenesis in order to confirm the locations of these putative NLS and NES sequences. Leptomycin B (LMB) can be used to identify the presence of a NES motif in a protein because it inhibits the CRM1 pathway and such a result has been found for VP3 of CAV. Using LMB, our results suggest that VP2 does not contain a functional NES and also that VP2 is CRM1 independent. Additionally, using a co-immunoprecipitation assay, we also found that VP2 associates with MCM3 and that this interaction does not require DSP activity.

## Results

### Localization of VP2 in mammalian cells

**Figure 1:**
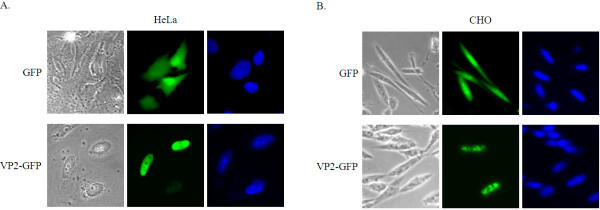
**VP2 protein is localized to the nucleus of the mammalian cells**. HeLa (**A**) and CHO (**B**) cells both were transfected with GFP and VP2-GFP expressing plasmids (green). At 48 h post-transfection, the cells were fixed and stained with DAPI (blue). The distribution of GFP and VP2-GFP in the cells were followed by microscopy (phase) and fluorescence microscopy

In previous studies, it has been shown by indirect immunoperoxidase staining that VP2 is localized within the nuclei of MDCC-MSB 1 cells infected with CAV [16]. In 2007, Lacorte et al. reported that transient expression of GFP-VP2 could be observed throughout the nucleoplasm in plant cells [25]. In the present study, we constructed expression plasmids of VP2-GFP in order to identify the nuclear localizing characteristics in cells. After transient transfection, the bright fluorescence of VP2-GFP was observed mostly in the nucleus of both HeLa and CHO cells. In contrast, when GFP alone was introduced, not only the nucleus but also the cytoplasm was fluorescent. This indicates that a functional NLS was present in VP2 as suggested by previously (Figure 1A and 1B).

### Using bioinformatics to predict the NLS and NES containing regions

**Figure 2:**
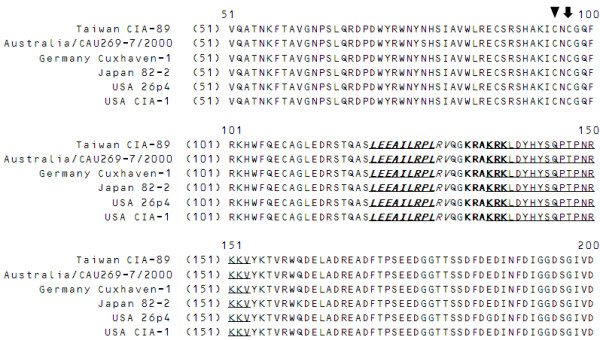
**Analysis and predication of NLS and NES motifs present in the VP2 amino acid sequence**. The various VP2 amino acid sequences (51 to 200) from different CAV isolates were aligned as described in the Materials and Methods. The putative NLS motifs (BiNLS1: under line and NLS2: bold words) and NES motifs (under line, bold, shadow and Italic) are shown. The cysteine residues at positions 95 and 97 in the catalytic motif of VP2 are also indicated by an arrow and an arrow head, respectively

**Figure 3:**
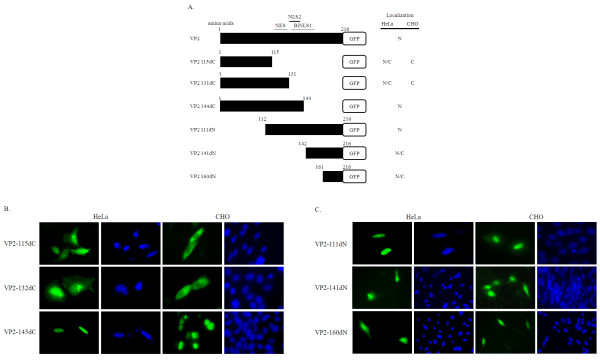
**Mutant constructs of VP2-GFP that were used in this study**. (**A**) Truncated fragments of the VP2 (black bars) encoding constructs fused with GFP (white bars) were used in this study. The right panels indicate the localization patterns in cells. GFP distribution in the cytosol is indicated by C; GFP signal in nucleus that is stronger than that in the cytosol is indicated by N; GFP detected in both the cytosol and nucleus is indicated by N/C. The putative NLS motifs (BiNLS1 and NLS2) and the weak NES motif are shown. The amino acids present in the constructs are also indicated. (**B**) Subcellular localization of C-terminal deleted VP2-GFP constructs (115dC, 132dC and 145dC) in HeLa and CHO cells (green). (**C**) Subcellular localization of N-terminal deleted VP2-GFP constructs (111dN, 141dN and 160dN) in HeLa and CHO cells (green). All cells were fixed and stained with DAPI (blue)

In order to identify if there are any NLS or NES motifs in VP2, we first compared the amino acid sequence of VP2 (Taiwan CIA-89) with a range of other CAV isolates to explore the protein's sequence divergence. The various VP2 sequences of the different CAV isolates were obtained from the UniProtKB database (http://www.uniprot.org/). Based on the sequence alignment, the VP2 proteins of these CAV isolates are highly conserved compared to strain CIA-89 from Taiwan. Therefore, the full length of amino acid sequence of VP2 (Taiwan CIA-89) was used and examined in order to predict NLS sequences using the WoLF PSORT and NLStradamus programs (Figures 2 and 3A). A bipartite NLS motif (named BiNLS1) was predicted by the WoLF PSORT program, with the putative motif position spanned amino acid residues from 136 to 153 (under line). However, a monopartite NLS motif (named NLS2) was also predicted by NLStradamus at a prediction cutoff value at 0.5 and this motif was located from amino acid residues from 133 to 138 (bold). Based on the results of bioinformatics analysis, VP2 was predicted to containing two possible NLS motifs. In addition, the NES motif prediction was performed by NetNES 1.1 Server (Figures 2 and 3A). A putative NES from amino acid residues 120 to 128 (under line, bold, shadow and Italic) was pinpointed. However, the expected value for NES prediction was lower than threshold expected value of this program, which suggests that there is a low probability of a NES motif existing within VP2. Based on these results, further investigations were needed to elucidate whether or not a NES is functional in VP2.

### Identification of BiNLS1 function in VP2

As mentioned above, two putative NLS motifs, a bipartite BiNLS1 and a monopartite NLS2 were predicted to be present in VP2. To determine the exactly site of the NLS motif in VP2, we constructed a full length clone and six deletion clones of VP2 fused with GFP at the C-terminus (Figure 3A). The subcellular locations of these expressed constructs in the transfected cells based on the GFP distribution pattern at 48 h post-transfection were examined. The truncated C-terminal deletion of VP2-GFP fusion proteins were VP2 115dC, VP2 132dC, and VP2 145dC. The N-terminal deletion mutants were VP2 111dN, VP2 141dN, and VP2 160dN. In Figure 3B, two of the C-terminal deletions, VP2 115dC, and VP2 132dC showed fluorescence distributed in both the nucleus and cytoplasm of HeLa cells (Figure 3B), whereas the bright fluorescence of these two truncations was predominantly distributed in the cytoplasm of CHO cells (Figure 3B). Fluorescence due to VP2 145dC was localized in the nucleus of both HeLa and CHO cells (Figure 3B). Furthermore, VP2 111dN was also localized to the nucleus of HeLa and CHO cells (Figure 3C). In contrast, fluorescence by VP2 141dN and VP2 160dN was found to have a very similar pattern to that of GFP in both the nucleus and cytoplasm of HeLa and CHO cells (Figure 3C).

**Figure 4:**
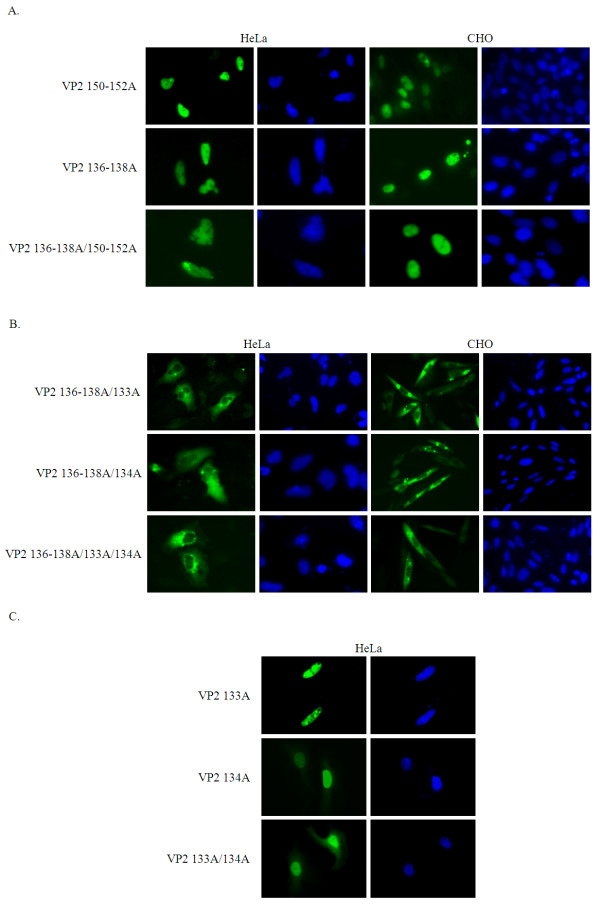
**Identification of the NLS motifs (BiNLS1 and NLS2) in VP2**. (**A**) Substitutive mutagenesis was used to create three constructs, VP2 150-152 (RKK to AAA), VP2 136-138 (KRK to AAA), as well as double and triple mutations, within the BiNLS1 region of VP2 (also saw in Table 1). The subcellular localization of these mutants (green) were examined by fluorescent microscopy in HeLa and CHO cells. (**B**) To further identify the functional NLS motif, K133 and R134 were replaced by alanine. The mutants (VP2 136-138A/134A, VP2 136-138A/133A and VP2 136-138A/133A/134A) were then expressed and examined by fluorescent microscopy (green). All cells were fixed and stained with DAPI (blue). (**C**) Identification of NLS2 in VP2. The point mutations VP2 133A, VP2 134A and VP2 133A/134A were transfected into HeLa cells. After 48 h post-transfection, the distributions of GFPs (green) were monitored by fluorescence microscopy. The cells were fixed and stained with DAPI (blue)

On comparing the fluorescence distribution patterns of VP2 111dN and VP2 141dN in mammalian cells, we found that the C-terminus part of BiNLS1 from VP2 did not seem to contain a nuclear import function. To support this finding, we used site directed mutagenesis to create a series of BiNLS1 mutants, namely VP2 150-152A (R150A, K151A and K152A), VP2 136-138A (K136A, R137A, and K138A) and VP2 136-138A/150-152A, as shown in Table 1. All of these mutants were able to express and were localized to the nucleus of HeLa and CHO cells (Figure 4A). The results indicated that NLS motif did not include the whole putative region of BiNLS1 as predicted by bioinformatics.

**Table 1:** Intracellular localization of VP2 and various mutants of the NLS motif

Name of mutants	Localization^a^	NLS motifs of amino acids sequence^b, c^
VP2-GFP	N	**K^133^R^134^A^135^K^136^R^137^K^138^**L^139^D^140^Y^141^H^142^Y^143^S^144^Q^145^P^146^T^147^P^148^N^149^R^150^K^151^K^152^

VP2 150-152A	N	**K^133^R^134^A^135^K^136^R^137^K^138^**L^139^D^140^Y^141^H^142^Y^143^S^144^Q^145^P^146^T^147^P^148^N^149^A^150^A^151^A^152^

VP2 136-138A	N	**K^133^R^134^A^135^A^136^A^137^A^138^**L^139^D^140^Y^141^H^142^Y^143^S^144^Q^145^P^146^T^147^P^148^N^149^R^150^K^151^K^152^

VP2 136-138A/150-152A	N	**K^133^R^134^A^135^A^136^A^137^A^138^**L^139^D^140^Y^141^H^142^Y^143^S^144^Q^145^P^146^T^147^P^148^N^149^A^150^A^151^A^152^

VP2 136-138A/133A	C	**A^133^R^134^A^135^A^136^A^137^A^138^**

VP2 136-138A/134A	C	**K^133^A^134^A^135^A^136^A^137^A^138^**

VP2 136-138A/133A/134A	C	**A^133^A^134^A^135^A^136^A^137^A^138^**

VP2 133A	N	**A^133^R^134^A^135^K^136^R^137^K^138^**

VP2 134A	N	**K^133^A^134^A^135^K^136^R^137^K^138^**

VP2 133A/134A	N	**A^133^A^134^A^135^K^136^R^137^K^138^**

a. The N and C indicate nuclear and cytoplasm, respectively.

b. The BiNLS1 and NLS2 motifs are indicated by underlining and bold, respectively.

c. The amino acids of BiNLS1 are shown from K136 to K152 and of NLS2 are shown from K133 to K138.

### The nuclear localization signal is within the NLS2 motif of VP2

**Figure 5:**
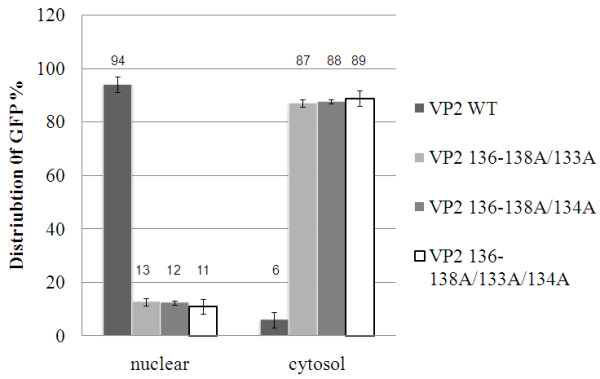
**The distribution of VP2-GFP and mutants in cytoplasm and nucleus of cells**. VP2-GFP and various mutants (VP2 136-138A/134A, VP2 136-138A/133A and VP2 136-138A/133A/134A) were transfected into CHO cells. After 48 h transfection, all cells were fixed and stained with DAPI. The distribution of GFP was monitored by fluorescence microscopy. In VP2-GFP and mutants, twenty cells were measured the distribution of GFP signals from cytoplasm and nucleus using Alpha View^® ^Software (Alpha Innotech Corporation)

Next, we focused on NLS2 motif in order to characterize the NLS motif within VP2. The VP2 132dC mutant produced fluorescence that was distributed across both the nucleus and cytoplasm of HeLa cells and in the cytoplasm of CHO cells. In contrast, the fluorescence produced by VP2 145dC was localized to the nucleus of HeLa and CHO cells. These results indicate that NLS motif of VP2 would seem to span amino acid residues 132 to 144. This region contains the NLS2 motif, which covers amino acid residues 133 to 138. We changed the positive charge of amino acids from 136 to 138 residues to alanine and measured the distribution of GFP. We found that the nuclear localization of GFP fluorescence of the VP2 136-138A mutants within this region was not abolished. However, our results indicated the presence of critical amino acid residues within the NLS motif of VP2 that play an essential role in nuclear transport. Specifically, alanine substitution mutagenesis at positions K133A and R134A of VP2 and of the VP2 136-138A clone was carried out to further characterize the nuclear localization motif and the results were summarized in Table 1. These results were shown that nuclear transportation of VP2 was obviously disrupted in three alanine substitution constructs, namely VP2 136-138A/133A, VP2 136-138A/134A, and VP2 136-138A/133A/134A (Figure 4B) but not in VP2 133A, VP2 134A and VP2 133A/134A (Figure 4C). The GFP signal distributions of VP2-GFP and its mutants in CHO cells are shown in Figures 1B and 4B and these were analyzed quantitatively by using Alpha View^® ^Software (Figure 5). VP2-GFP was 94% of the time in nucleus and 6% in cytosol. The nuclear presence of the VP2 136-138A/133A, VP2 136-138A/134A, and VP2 136-138A/133A/134A mutant was reduced to 13%, 12% and 11%, respectively, with most of GFP signal accumulated in the cytoplasm. These findings demonstrate that NLS2 is a functional NLS motif in VP2 and that amino acid residues K133 and R134 are also important to this functionality (Figures 4B and 5).

### VP2 is a CRM1 independent protein

Typical NES motifs have been found in the various viral and cellular proteins and are involved in transportation between the nucleus and the cytoplasm [26]. LMB has been shown to interfere with the CRM1-NES interaction and can be used to verify the functionality of any NES motif. A weak putative NES motif spanning amino acid residues from 120 to 128 of VP2 was predicted earlier (Figure 2). However, the prediction score was lower than the program threshold. The weak putative NES motif was investigated using the truncated mutants shown in Figure 3A together with LMB (+) (20 ng/ml) and LMB (-) (PBS) buffers, which were added to cells at 48 h post-transfection and incubated for 1 h [27,28]. VP3-GFP was used as a positive control because it has been reported to be a CRM1 dependent protein and contains a NES motif [29,30]. The distribution of fluorescence was then observed (see Additional file 1) and the results demonstrated that, while LMB was able to affect the nuclear export of VP3, it had no effect on VP2. Therefore it would seem that VP2 nuclear export is a CRM1 independent process and that there is no functional NES motif present in VP2.

### VP2 binds to chromatin

**Figure 6:**
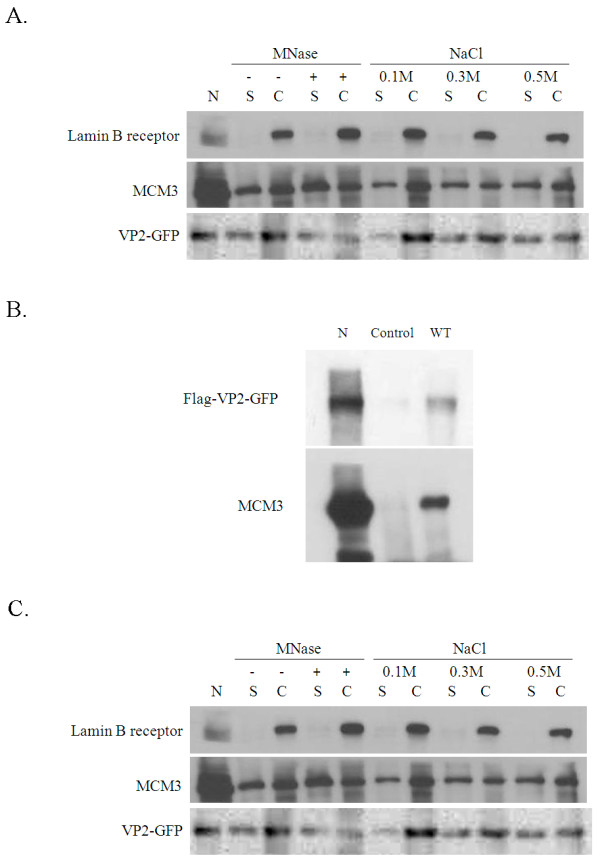
**VP2 binds to chromatin and interacts with MCM3 but this does not require dual phosphatase activity**. (**A**) The soluble (S) and chromatin (C) fractions were prepared by using CSK buffer containing 0.5% Triton X-100 in CHO cells of transfected with VP2-GFP plasmid. The fractions were treated with 0 U (-) and 150 U (+) of MNase or the indicated concentrations of NaCl. Nuclear extracts (N) containing VP2-GFP were as positive control and all fractions were subjected to immunoblotting against with lamin B receptor, MCM3, and VP2 antibodies. (**B**) At 48 h post-transfection, the GFP (as Control) and Flag-VP2-GFP (as wild-type; WT) were found in the CHO cells. Next the lysates were immunoprecipitated using Flag M2 beads and immunoblotted against VP2 and MCM3 antibodies. (**C**) The WT and mutants (C95S, C97S, and C95S/C97S) of the Flag-VP2-GFP in CHO cells were also examined at 48 h post-transfection. The cell lysates were immunoprecipitated using Flag M2 beads and immunoblotted against with VP2 and MCM3 antibodies. The arrow head was indicated a none-specific band. The nuclear extracts containing VP2-GFP were designated as N

As previously reported and confirmed by this study, VP2-GFP predominantly is localized in nucleus (Figure 1). In order to further investigate this localization, CHO cells were transfected with VP2-GFP plasmid and the resulting cell extract separated into soluble and chromatin fractions. As is shown in Figure 6A, VP2-GFP could be detected in both the soluble and chromatin fractions. The chromatin fraction was further digested with micrococcal nuclease (MNase). Like MCM3, VP2-GFP was only partial dissociated from the chromatin fraction after treatment with MNase, which suggested that VP2 is a chromatin-bound protein and MNase-resistant. The chromatin fraction was also treated with different concentration of NaCl. The result is shown in Figure 6A, whereby VP2-GFP was found to be slightly dissociation at 0.3 M NaCl and 0.5 M NaCl, but a significant amount of remaining protein remained bound to the chromatin. When tested under similar conditions, the lamin B receptor, a nuclear matrix protein, was not extracted by 0.3 M NaCl and 0.5 M NaCl. Therefore, these results strongly suggest that VP2, like MCM3, is a chromatin binding protein, but was not a nuclear matrix protein.

### The DSP activity of VP2 is not required for protein-protein interaction with MCM3

In a previous study, the DSP activity of VP2 was shown to be needed for virus replication in the cell [28]. In order to address what proteins in the various DNA replication complexes interacts with VP2 in cells, an independent assay involving immunoprecipitation using anti-Flag M2 beads against Flag-VP2-GFP was employed. In the control experiments, GFP expressing plasmids were transfected into cells and it was found that no none-specific proteins were brought down by the Flag M2 beads using Western blot detection (Figure 6B, lane Control). On the other hand, Flag-VP2-GFP was co-immunoprecipited with MCM3 protein by the Flag M2 beads (Figure 6B, lane WT) when compared to the protein band in the Flag-VP2-GFP nuclear extract (Figure 6B, lane N). In contrast, other components of DNA replication complexes such as CDC7, PCNA and condensin SMC2, as well as the core histones such as H2B, were not co-immunoprecipited with VP2 using CHO cell extracts (see Additional file 2). The above results confirmed that VP2 associates with MCM3 when it binds to chromatin.

To identify whether the dual phosphatase activity of VP2 was needed for association with MCM3, we performed a site directed mutagenesis assay targeting position C95 and C97 of VP2 in order to create the single mutants C95S and C97S and the double mutant C95S/C97S, which have either partial or complete disruption of the DSP activity associated with VP2 [17]. Co-immunoprecipitation assays were carried out and, the results are shown in Figure 6C, MCM3 was co-immunoprecipitated by the three mutants of VP2 protein in a similar manner to the wild type protein, which shows that VP2 binding to MCM3 does not require dual-phosphatase activity (Figure 6C).

## Discussion

CAV is an important avian pathogen worldwide and causes major economic damage throughout the poultry industry. Three major proteins are encoded by this virus, namely the capsid protein VP1, the nonstructural protein VP2, and the apoptin VP3. However, up to the present, the exactly functions of these three proteins in cell are still poorly understood. In previous studies, the expression of VP2 was found to be nuclear in plant cells [25]. This result is similar to that of the present study, where, VP2 was found to accumulate in the nucleus of both HeLa and CHO cells. Our results, both by bioinformatics and site-directed mutagenesis, support the hypothesis that VP2 contain a NLS and named as NSL2. NLS2 was shown to be a functional NLS that allowed transfer of VP2 into nucleus. This is similar to VP3, which has two functional NLS motifs that are also used for nuclear localization. The single VP2 NLS motif spans amino acid residues 133 to 138. Within this motif, we also found that two amino acids with positive charges, 133R and 134 K were important to allowing VP2 protein to shuttle from the cytoplasm to the nucleus. To the best of our knowledge, this is the first report identifying a NLS motif in the VP2 of CAV.

Subsequent sequence analysis showed that VP2 might contain a putative CRM1-mediated NES motif that stretched from amino acid 120 to amino acid 128, however the scores for this motif was below the program threshold. In order to confirm that a NES motif really existed in VP2, we used fusion protein constructs to investigate nuclear transportation in the presence of LMB assay (Figures 3A and see Additional file 1). A control protein, namely VP3, also named apoptin, which contains a classical leucine-rich NES motif and has been previously described as sensitive to LMB treatment was included in the study [29,30]. Our results confirmed that the cytoplasm and nuclear distribution of the various truncated VP2 mutants was not obviously changed in pattern after treatment with LMB. This supports two possibilities; firstly, that the nuclear export pathway (using NES motif) of VP2 might not exist and/or, secondly, that a distinct and CRM1 dependent pathway was adopted by VP2 for nuclear export.

VP2 has been shown to contain dual phosphatase activity and this activity is required for CAV replication [17,31]. However, in this context, very little is known about the role this protein plays in the regulation of viral DNA replication. When cells are infected with CAV, VP2 is expressed and accumulates, reaching a detectable level at 12 h post-infection [8,16]. In contrast, VP1 has been found to accumulate to a higher level at 30 h post-infection. Amino acids sequence analysis of CAV VP1 was revealed that the N-terminal region show similarity to protamines [11], this supports the hypothesis that VP1 has DNA-binding functionality [32]. The N and C-terminal domains of VP3 separately bind to DNA and indicating the presence of multiple independent binding sites [33]. However, VP2 protein has no obvious DNA binding characteristics. In this study, VP2 of CAV is shown to be localized to the nucleus in two cell lines (Figure 1) and also to bind to chromatin (Figure 6). The latter finding was confirmed by examining the nuclease-resistant fraction and by treating with high salt. VP2 is similar to MCM3, a component of various MCM2-7 complexes, and other nuclease-resistant chromatin bound proteins such as CDC6 [18], minichromosome maintenance proteins (MCMs) [34], and PCNA [35], all of which have chromatin-bound characteristics. This suggests that the early expression of VP2 might involve DNA replication with VP2 interacting with the prereplication complex (Figure 6B). The association of VP2 with MCM3 (and perhaps other members of a MCM2-7 complex) may facilitate the DNA replication of CAV. However, it is still unknown whether VP2 is able to bind to DNA directly and this will need further investigation. Moreover, VP2's association with MCM3 does not require dual phosphatase activity (Figure 6C) and therefore, the relevance of VP2's DSP activity to the live cycle of CAV also needs further investigation.

Rep, a viral protein with replicase activity involved in regulating rolling-circle replication (RCR), has been found in most circovirus members of the family Circoviridae [36]. CAV seems to lack a Rep protein. However, some studies have proposed that the C-terminus of CAV VP1 contains a highly conserved RCR motif and that this may play a role in regulating the RCR reaction [32]. This RCR-regulating function of VP1 seems to be very similar to that of the Rep protein. Therefore, VP1 may interact with VP2 to form a nucleoprotein complex and, in addition, the genomic DNA of the virus might also be coupled with VP1 and VP2 in order to regulate the RCR reaction during the early stages of infection. However, these hypotheses need to be investigated in terms of how the MCMs interact with the VP2 of CAV.

## Conclusions

We have demonstrated that the VP2 of CAV contains a functional NLS motif that spans amino acids 133 to 138 of the protein. In addition to having a NLS, VP2 is also a chromatin binding protein similar to members of the MCM2-7 complex. Moreover, VP2 associates with MCM3 in cells based on co-immunoprecipitation analysis. Taken together, these findings suggest that VP2 may be part of a DNA pre-replication complex.

## Methods

### Antibodies

All primary anti-human and mouse antibodies used for immunoblotting were purchased from commercial companies. Rabbit polyclonal anti-human and mouse antibodies were obtained as follows: SMC2, CDC7, and H2B from Santa Cruz Biotechology, USA; MCM3 from Bethyl Laboratories, USA; PCNA from Epitomics, USA and rabbit monoclonal anti-human and mouse lamin B receptor antibody from Abcam, USA. The anti-VP2 of CAV polyclonal antibody was prepared by immunizing rabbits using VP2 of CAV expressed in *E. coli *[37]. The secondary antibodies for Western blotting were as follows: the goat anti-mouse IgG and goat anti-rabbit IgG conjugated HRP, which were both obtained from Santa Cruz Biotechology, USA.

### Construction of plasmids

Various plasmids, such as pcDNA3.1 VP2-GFP, pcDNA3.1 Flag-VP2-GFP and pcAcGFP1-N1 VP3-GFP, were constructed as described below. The full-length of VP2, Flag-VP2 and VP3 were amplified by PCR using high fidelity Platinum Taq DNA polymerase (Invitrogen, USA) from pGEX-6P-1-VP2 [38] and pGEX-6P-1-VP3 (data not shown) using the primers: forward VP2 1-18 EcoRI 5' TGGAATTCATGCACGGGAACGGCGGA3' or VP2 Flag EcoRI 5' AGGATCCATGGATTACAAGGATGACGACGATAAGGAATTCATGCACGGGAACGGCGGACA 3' and reverse VP2 657 XhoI 5' TCCTCGAGCACTATACGTACCGG 3' for VP2 and Flag-VP2 in addition to forward VP3 NheI 5' AGCTAGCATGAACGCTCTCCAAGAAG 3' and reverse VP3 XhoI 5' TCCTCGAGCAGTCTTATACACCTTCT 3' for VP3. The PCR products were then ligated into the yT&A vector (Yeastern Biotech, Taiwan). The *EcoRI*/*XhoI *fragments containing VP2 and Flag-VP2 or *NheI*/*XhoI *fragments containing VP3 were released from the yT&A vector and ligated into pcDNA3.1-GFP, which was a kind gift from Prof. Min-Ying Wang (the Graduate Institute of Biotechnology, National Chung Hsing University, Taichung, Taiwan) and pAcGFP1-N1 (Clontech, USA). The truncated and point mutations of the VP2-GFP constructs were generated in a similar manner to that described above and the primers are summarized in Table 2. Single site directed and multiple site directed mutagenesis were carried out by PCR using PfuUltra™ High-Fidelity DNA Polymerase (Stratagene, USA) in addition to using pcDNA3.1 VP2-GFP and pcDNA3.1 Flag-VP2-GFP as the template with the primers in Table 2.

**Table 2:** The primers used to create the various truncated, single and multiple mutants by PCR in this study

Primer name	Type	Length	Sequence (5'-3')
VP2 111 N del EcoRI	Forward	26-mer	TGGAATTCATGGAGGACCGATCAACC

VP2 141 N del EcoRI	Forward	26-mer	AGGAATTCATGCACTACTCCCAGCCG

VP2 160 N del EcoRI	Forward	26-mer	AGGAATTCATGGACGAGCTCGCAGAC

VP2 115 C del XhoI	Reverse	24-mer	TCCTCGAGTGATCGGTCCTCAAGT

VP2 132 C del XhoI	Reverse	23-mer	TCCTCGAGACCCTGTACTCGGAG

VP2 145 C del XhoI	Reverse	26-mer	TCCTCGAGCTGGGAGTAGTGGTAATC

VP2 136-138A	Forward	27-mer	AAACGAGCTGCTGCTGCTCTTGATTAC

VP2 136-138A	Reverse	27-mer	GTAATCAAGAGCAGCAGCAGCTCGTTT

VP2 150-152A	Forward	39-mer	ACCCCGAACGCAGCAGCAGTGTATAAGACTGTAAGATGG

VP2 150-152A	Reverse	39-mer	CCATCTTACAGTCTTATACACTGCTGCTGCGTTCGGGGT

VP2 136-138A/134/A	Forward	27-mer	GTACAGGGTAAAGCTGCTGCTGCTGCT

VP2 136-138A/134/A	Reverse	27-mer	AGCAGCAGCAGCAGCTTTACCCTGTAC

VP2 136-138A/133/A	Forward	27-mer	GTACAGGGTGCTCGAGCTGCTGCTGCT

VP2 136-138A/133/A	Reverse	27-mer	AGCAGCAGCAGCTCGAGCACCCTGTAC

VP2136-138A/133A/134/A	Forward	27-mer	GTACAGGGTGCTGCTGCTGCTGCTGCT

VP2 136-138A/133A/134/A	Reverse	27-mer	AGCAGCAGCAGCAGCAGCACCCTGTAC

VP2 133A	Forward	28-mer	GTACAGGGTGCTCGAGCTAAAAGAAAGC

VP2 133A	Reverse	28-mer	GCTTTCTTTTAGCTCGAGCACCCTGTAC

VP2 134A	Forward	28-mer	GTACAGGGTAAAGCTGCTAAAAGAAAGC

VP2 134A	Reverse	28-mer	GCTTTCTTTTAGCAGCTTTACCCTGTAC

VP2 133A/134A	Forward	28-mer	GTACAGGGTGCTGCTGCTAAAAGAAAGC

VP2 133A/134A	Reverse	28-mer	GCTTTCTTTTAGCAGCAGCACCCTGTAC

VP2 C95S	Forward	20-mer	CGCTAAGATCAGCAACTGCG

VP2 C95S	Reverse	22-mer	CGCAGTTGCTGATCTTAGCGTG

VP2 C97S	Forward	21-mer	ATCTGCAACAGCGGACAATTC

VP2 C97S	Reverse	24-mer	ATTGTCCGCTGTTGCAGATCTTAG

VP2 C95S/C97S	Forward	28-mer	CGCTAAGATCAGCAACAGCGGACAATTC

VP2 C95S/C97S	Reverse	28-mer	ATTGTCCGCTGTTGCTGATCTTAGCGTG

### Amino acid sequence analysis and predication

The amino acid sequences of VP2 from different isolates were identified by searching the UniProtKB database (available at http://www.uniprot.org/). The isolates analyzed were Taiwan CIA-89 (in the present paper), Australia/CAU269-7/2000 (accession number: Q9IZU7), Germany Cuxhaven-1(accession number: P69484), Japan 82-2 (accession number: P54093), USA 26p4 (accession number: P54092), and USA CIA-1(accession number: P69485). The amino acid sequences were then aligned and analyzed using program of Biology Workbench 3.2 (San Diego Supercomputer Center; SDSC). The putative NES motif was predicted by the NetNES 1.1 Server [39] while the NLS was predicted by WoLF PSORT [40] and NLStradamus [41].

### Cell culture, transfection and staining

Chinese hamster ovary (CHO) cells were grown in GIBCO^® ^Dulbecco's Modified Eagle Medium: Nutrient Mixture F-12 (DMEM/F-12) medium (Invitrogen, USA) supplemented with 10% (v/v) fetal bovine serum (FBS) (GIBCO/Invitrogen, USA), 100 units/ml penicillin, and 100 μg/ml streptomycin. HeLa cells were grown in Dulbecco's minimal essential medium (DMEM) (Invitrogen, USA) supplemented with 10% (v/v) FBS, 100 units/ml penicillin, and 100 μg/ml streptomycin. In order to determine the localization of VP2-GFP and the various mutant proteins, pcDNA3.1 VP2-GFP and the various mutant plasmids were transfected into HeLa and CHO cells using TurboFect™ (Fermentas, Canada) by following the manufacturer's instructions. The plasmid of pAcGFP-N1 VP3-GFP was also transfected as a positive control for the LMB treatment assay. HeLa or CHO cells were seeded at a density of 2 × 10^4 ^or 8 × 10^4 ^cells per well in 24 well culture plates. Forty-eight hours after transfection, aliquots of the cells, underwent replacement with fresh medium containing 20 ng/ml LMB (+) (Calbiochem, Germany) [27,28] or phosphate-buffered saline (PBS) buffer as the LMB (-) control. This was in order to test the nuclear export signals of VP2 and VP3. All cells were fixed using 4% paraformaldehyde solution. After washing three times with 1× PBS, the cells were incubated with 1 × PBS containing 0.25% Triton X-100 for 10 min and stained with 1 μg/ml DAPI (Sigma, USA). GFP fluorescence and DAPI images were captured using a ZEISS AXIOVERT 200 microscope equipped with a 40 objective and an AxioCam HRm CCD camera. Image processing was done using Photoshop. The Alpha View^® ^Software (Alpha Innotech Corporation, USA) was used to calculate the distribution of VP2-GFP and mutants across the cytosol and nucleus in the cells.

### Cell fractionations

Chromatin fractions were prepared by minor modifications of a procedure described previously [42]. After transfection with VP2-GFP plasmid, the CHO cells were lysed in cytoskeleton (CSK) buffer containing 0.5% Triton X-100, 1 mM ATP, 1 mM dithiothreitol, and protease inhibitors (Sigma, USA) for 30 min on ice. The protein extract was then centrifuged at 1500 *g *for 5 min at 4°C. The supernatant was collected and labeled as the soluble fraction (S). The pellet which was chromatin (C) was washed once with CSK buffer for 5 min on ice, then centrifuged at 1500 *g *for 5 min at 4°C, and resuspended in SDS sample buffer. For micrococcal nuclease (MNase) (Fermentas, Canada) or NaCl treatment, the chromatin was resuspended in 100 μl of CSK buffer supplemented with 0.1 M, 0.3 M of NaCl or 0 U (-) and 150 U (+) MNase with 2 mM CaCl_2 _and incubated for 30 min at 37°C. After incubation, the chromatin and soluble supernatant were separated by centrifugation. Chromatin and soluble solutions were resuspended and boiled in SDS sample buffer for immunoblotting.

### Co-immunoprecipitation

After transient transfection with pcDNA 3.1 Flag-VP2-GFP and the various mutants, CHO cells were harvested and lysed using Nonidet P-40 (NP-40) lysis buffer (10 mM HEPES, pH 8.0, 0.2% NP-40, 150 mM NaCl, 1 mM EGTA, 5 mM MgCl_2 _and 10% glycerol) containing a cocktail of protease inhibitors (Sigma, USA). After 30 min on ice, the lysates were centrifuged at 13200 rpm for 5 min at 4°C. For co-immunoprecipitation, the 500 μg/ml cell extracts were incubated with 2 μg of anti-Flag M2 beads (Sigma, USA) for 16 h at 4°C. The immunoprecipitate was then washed three times with lysis buffer, resuspended in 20 μl of SDS sample buffer, and the samples were heated for 5 min at 100°C. The soluble proteins were resolved by SDS-PAGE for Western blotting.

## Competing interests

MSL, JHC and YYL are inventors on a patent submission entitled: A method for Protein Translocating into Nucleus Using A Specific Peptide Sequence for. The other authors declare no competing interests.

## Authors' contributions

JHC and MSL participated in the design and coordination of the study, carried out the study, performed the bioinformatics analysis, and wrote the manuscript. SCS and YYL participated in the design of the primers, experiments for clone and wrote the manuscript. MSL and WHS participated in experiments for clone. HJC participated in the bioinformatics analysis. All authors read and approved the final manuscript.

## Supplementary Material

Additional file 1 **The effect of LMB treatments on the various mutants of VP2**. The truncated mutants of VP2 in Figure 3A were all treated with LMB (+) (20 ng/ml) and LMB (-) (PBS buffer) for 1 h at 37°C. VP3-GFP is LMB sensitive and was used as a positive control. The distribution of GFP was monitored by fluorescence microscopy. Click here for file

Additional file 2 **Identification of protein-protein interactions with VP2 surveyed by co-immunoprecipitation**. At 48 h post-transfection with plasmids encoding GFP (as the Control) or Flag-VP2-GFP (WT), cell lysates were immunoprecipitated by Flag M2 beads and immunoblotted against VP2, MCM3, CDC7, SMC2, PCNA, and H2B antibodies. The nuclear extracts containing VP2-GFP are designated as N. Click here for file

## References

[B1] Yuasa NTT Yoshida I Isolation and some characteristics of an agent inducing anemia in chicks Avian Dis 1979 23 366 385 10.2307/1589567

[B2] Noteborn MH Chicken anemia virus induced apoptosis: underlying molecular mechanisms Vet Microbiol 2004 98 89 94 10.1016/j.vetmic.2003.10.003 14741120

[B3] McNulty MS Connor TJ McNeilly F Spackman D Chicken anemia agent in the United States: isolation of the virus and detection of antibody in broiler breeder flocks Avian Dis 1989 33 691 694 10.2307/1591146 2533491

[B4] Yuasa NNT Furuta K Yoshida I Maternal antibody and its effect on the susceptibility of chicks to chicken anemia agent Avian Dis 1980 24 197 201 10.2307/1589779

[B5] Lucio B Schat KA Shivaprasad HL Identification of the chicken anemia agent, reproduction of the disease, and serological survey in the United States Avian Dis 1990 34 146 153 10.2307/1591346 2108663

[B6] Yuasa N Imai K Watanabe K Saito F Abe M Komi K Aetiological examination of an outbreak of haemorrhagic syndrome in a broiler flock in Japan Avian Pathol 1987 16 521 526 10.1080/03079458708436401 18766640

[B7] Hsu JP Lee ML Lu YP Hung HT Hung HH Chein MS Chicken infectious anemia in layer J Chin Soc Vet Sci 2002 28 153 160

[B8] Schat KA Chicken anemia virus Curr Top Microbiol Immunol 2009 331 151 183 10.1007/978-3-540-70972-5_10 19230563

[B9] Lu YS Tsai HJ Kwang MJ Tseng CS Chicken infectious anemia in Taiwan: virus isolation and antibody survey Exp Rep TPRIAH 1993 29 81 89

[B10] Meehan BM Todd D Creelan JL Earle JA Hoey EM McNulty MS Characterization of viral DNAs from cells infected with chicken anaemia agent: sequence analysis of the cloned replicative form and transfection capabilities of cloned genome fragments Arch Virol 1992 124 301 319 10.1007/BF01309811 1605740

[B11] Claessens JA Schrier CC Mockett AP Jagt EH Sondermeijer PJ Molecular cloning and sequence analysis of the genome of chicken anaemia agent J Gen Virol 1991 72 Pt 8 2003 2006 1908516 10.1099/0022-1317-72-8-2003

[B12] Ducatez MF Owoade AA Abiola JO Muller CP Molecular epidemiology of chicken anemia virus in Nigeria Arch Virol 2006 151 97 111 10.1007/s00705-005-0609-7 16096706

[B13] Phenix KV Meehan BM Todd D McNulty MS Transcriptional analysis and genome expression of chicken anaemia virus J Gen Virol 1994 75 Pt 4 905 909 8151304 10.1099/0022-1317-75-4-905

[B14] Noteborn MH Kranenburg O Zantema A Koch G de Boer GF van der Eb AJ Transcription of the chicken anemia virus (CAV) genome and synthesis of its 52-kDa protein Gene 1992 118 267 271 10.1016/0378-1119(92)90198-X 1511899

[B15] Pallister J Fahey KJ Sheppard M Cloning and sequencing of the chicken anaemia virus (CAV) ORF-3 gene, and the development of an ELISA for the detection of serum antibody to CAV Vet Microbiol 1994 39 167 178 10.1016/0378-1135(94)90097-3 8203122

[B16] Douglas AJ Phenix K Mawhinney KA Todd D Mackie DP Curran WL Identification of a 24 kDa protein expressed by chicken anaemia virus J Gen Virol 1995 76 Pt 7 1557 1562 9049362 10.1099/0022-1317-76-7-1557

[B17] Peters MA Jackson DC Crabb BS Browning GF Chicken anemia virus VP2 is a novel dual specificity protein phosphatase J Biol Chem 2002 277 39566 39573 10.1074/jbc.M201752200 12151384

[B18] Mendez J Stillman B Chromatin association of human origin recognition complex, cdc6, and minichromosome maintenance proteins during the cell cycle: assembly of prereplication complexes in late mitosis Mol Cell Biol 2000 20 8602 8612 10.1128/MCB.20.22.8602-8612.2000 11046155 PMC102165

[B19] Noteborn MH Todd D Verschueren CA de Gauw HW Curran WL Veldkamp S Douglas AJ McNulty MS van Der EA Koch G A single chicken anemia virus protein induces apoptosis J Virol 1994 68 346 351 8254747 10.1128/jvi.68.1.346-351.1994 PMC236294

[B20] Kamada K Kuroishi A Kamahora T Kabat P Yamaguchi S Hino S Spliced mRNAs detected during the life cycle of Chicken anemia virus J Gen Virol 2006 87 2227 2233 10.1099/vir.0.81946-0 16847118

[B21] Koch G van Roozelaar DJ Verschueren CA van der Eb AJ Noteborn MH Immunogenic and protective properties of chicken anaemia virus proteins expressed by baculovirus Vaccine 1995 13 763 770 10.1016/0264-410X(94)00034-K 7483793

[B22] Noteborn MH Verschueren CA Koch G Van der Eb AJ Simultaneous expression of recombinant baculovirus-encoded chicken anaemia virus (CAV) proteins VP1 and VP2 is required for formation of the CAV-specific neutralizing epitope J Gen Virol 1998 79 Pt 12 3073 3077 9880024 10.1099/0022-1317-79-12-3073

[B23] Noteborn MH Verschueren CA van Ormondt H van der Eb AJ Chicken anemia virus strains with a mutated enhancer/promoter region share reduced virus spread and cytopathogenicity Gene 1998 223 165 172 10.1016/S0378-1119(98)00170-X 9858721

[B24] Adair BM Immunopathogenesis of chicken anemia virus infection Dev Comp Immunol 2000 24 247 255 10.1016/S0145-305X(99)00076-2 10717291

[B25] Lacorte C Lohuis H Goldbach R Prins M Assessing the expression of chicken anemia virus proteins in plants Virus Res 2007 129 80 86 10.1016/j.virusres.2007.06.020 17698236

[B26] Nigg EA Nucleocytoplasmic transport: signals, mechanisms and regulation Nature 1997 386 779 787 10.1038/386779a0 9126736

[B27] Davis M Hatzubai A Andersen JS Ben-Shushan E Fisher GZ Yaron A Bauskin A Mercurio F Mann M Ben-Neriah Y Pseudosubstrate regulation of the SCF(beta-TrCP) ubiquitin ligase by hnRNP-U Genes Dev 2002 16 439 451 10.1101/gad.218702 11850407 PMC155337

[B28] Kosugi S Hasebe M Tomita M Yanagawa H Nuclear export signal consensus sequences defined using a localization-based yeast selection system Traffic 2008 9 2053 2062 10.1111/j.1600-0854.2008.00825.x 18817528

[B29] Poon IK Oro C Dias MM Zhang J Jans DA Apoptin nuclear accumulation is modulated by a CRM1-recognized nuclear export signal that is active in normal but not in tumor cells Cancer Res 2005 65 7059 7064 10.1158/0008-5472.CAN-05-1370 16103052

[B30] Wang QM Fan GC Chen JZ Chen HP He FC A putative NES mediates cytoplasmic localization of Apoptin in normal cells Acta Biochim Biophys Sin (Shanghai) 2004 36 817 823 10.1093/abbs/36.12.817 15592649

[B31] Peters MA Jackson DC Crabb BS Browning GF Mutation of chicken anemia virus VP2 differentially affects serine/threonine and tyrosine protein phosphatase activities J Gen Virol 2005 86 623 630 10.1099/vir.0.80197-0 15722522

[B32] Todd D McNulty MS Adair BM Allan GM Animal circoviruses Adv Virus Res 2001 57 1 70 11680382 10.1016/s0065-3527(01)57000-1

[B33] Leliveld SR Dame RT Rohn JL Noteborn MHM Abrahams JP Apoptin's functional N- and C-termini independently bind DNA FEBS Letters 2004 557 155 158 10.1016/S0014-5793(03)01465-0 14741359

[B34] Edwards MC Tutter AV Cvetic C Gilbert CH Prokhorova TA Walter JC MCM2-7 complexes bind chromatin in a distributed pattern surrounding the origin recognition complex in Xenopus egg extracts J Biol Chem 2002 277 33049 33057 10.1074/jbc.M204438200 12087101

[B35] Rowles A Blow JJ Chromatin proteins involved in the initiation of DNA replication Curr Opin Genet Dev 1997 7 152 157 10.1016/S0959-437X(97)80123-2 9115430

[B36] Mankertz A Hillenbrand B Replication of porcine circovirus type 1 requires two proteins encoded by the viral rep gene Virology 2001 279 429 438 10.1006/viro.2000.0730 11162799

[B37] Lee MS Lien YY Feng SH Huang RL Tsai MC Chang WT Chen HJ Production of chicken anemia virus (CAV) VP1 and VP2 protein expressed by recombinant Escherichia coli Proc Biochem 2009 44 390 395 10.1016/j.procbio.2008.11.016

[B38] Huang CH Lai GH Lee MS Lin WH Lien YY Hsueh SC Kao JY Chang WT Lu TC Lin WN Chen HJ Development and evaluation of a loop-mediated isothermal amplification assay for rapid detection of chicken anaemia virus J Appl Microbiol 2010 108 917 924 10.1111/j.1365-2672.2009.04481.x 19737344

[B39] la Cour T Kiemer L Molgaard A Gupta R Skriver K Brunak S Analysis and prediction of leucine-rich nuclear export signals Protein Eng Des Sel 2004 17 527 536 10.1093/protein/gzh062 15314210

[B40] Horton P Park KJ Obayashi T Fujita N Harada H Adams-Collier CJ Nakai K WoLF PSORT: protein localization predictor Nucleic Acids Res 2007 35 W585 587 10.1093/nar/gkm259 17517783 PMC1933216

[B41] Nguyen Ba AN Pogoutse A Provart N Moses AM NLStradamus: a simple Hidden Markov Model for nuclear localization signal prediction BMC Bioinformatics 2009 10 202 10.1186/1471-2105-10-202 19563654 PMC2711084

[B42] Deng M Li F Ballif BA Li S Chen X Guo L Ye X Identification and functional analysis of a novel cyclin e/cdk2 substrate ankrd17 J Biol Chem 2009 284 7875 7888 10.1074/jbc.M807827200 19150984 PMC2658080

