# Supplementary figures and images for: Identification of the NLS and NES motifs of VP2 from chicken anemia virus and the interaction of VP2 with mini-chromosome maintenance protein 3
Source: BMC Vet Res. 2012 Feb 7;8:15. doi: 10.1186/1746-6148-8-15 (PMC3295642; doi:10.1186/1746-6148-8-15)

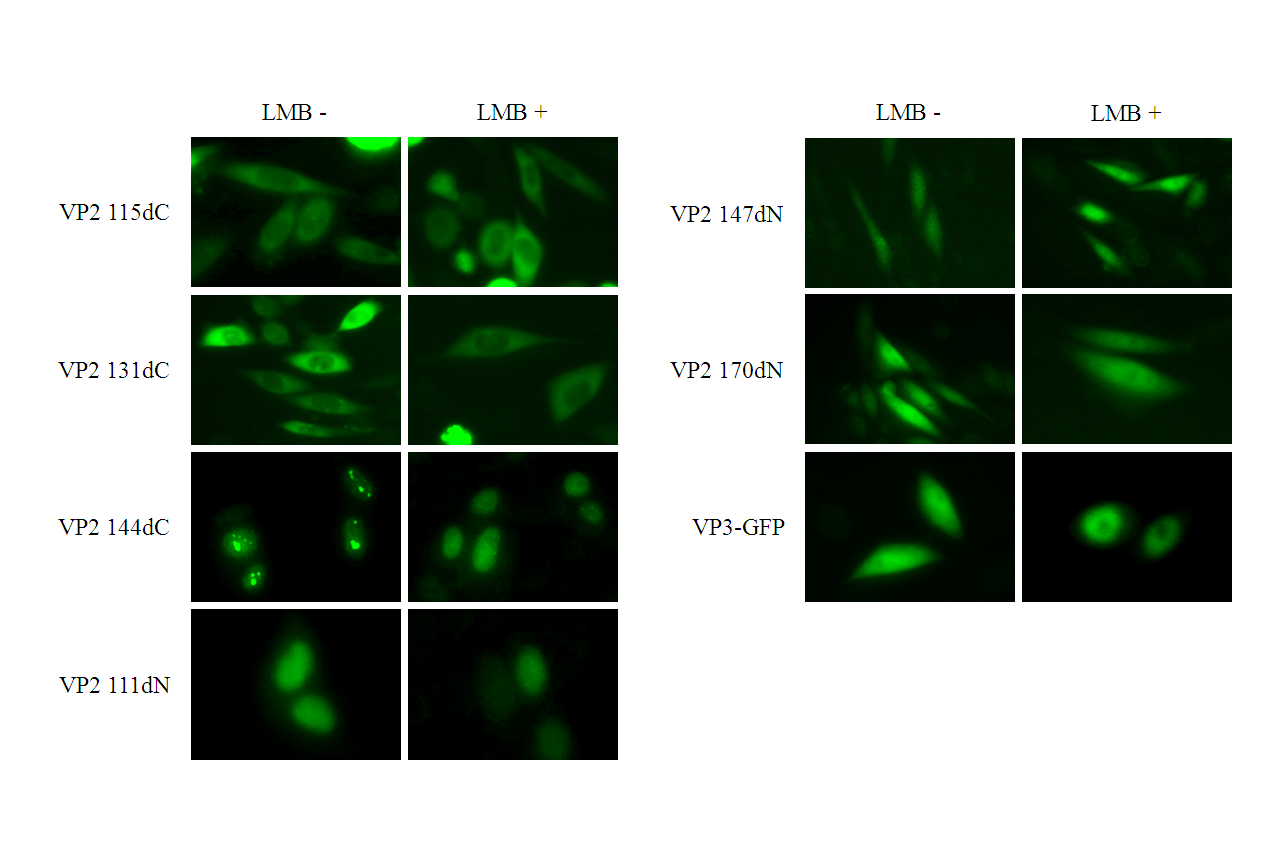

Supplement: Additional file 1 — The effect of LMB treatments on the various mutants of VP2. The truncated mutants of VP2 in Figure 3A were all treated with LMB (+) (20 ng/ml) and LMB (-) (PBS buffer) for 1 h at 37°C. VP3-GFP is LMB sensitive and was used as a positive control. The distribution of GFP was monitored by fluorescence microscopy. [file 1746-6148-8-15-S1.TIFF]

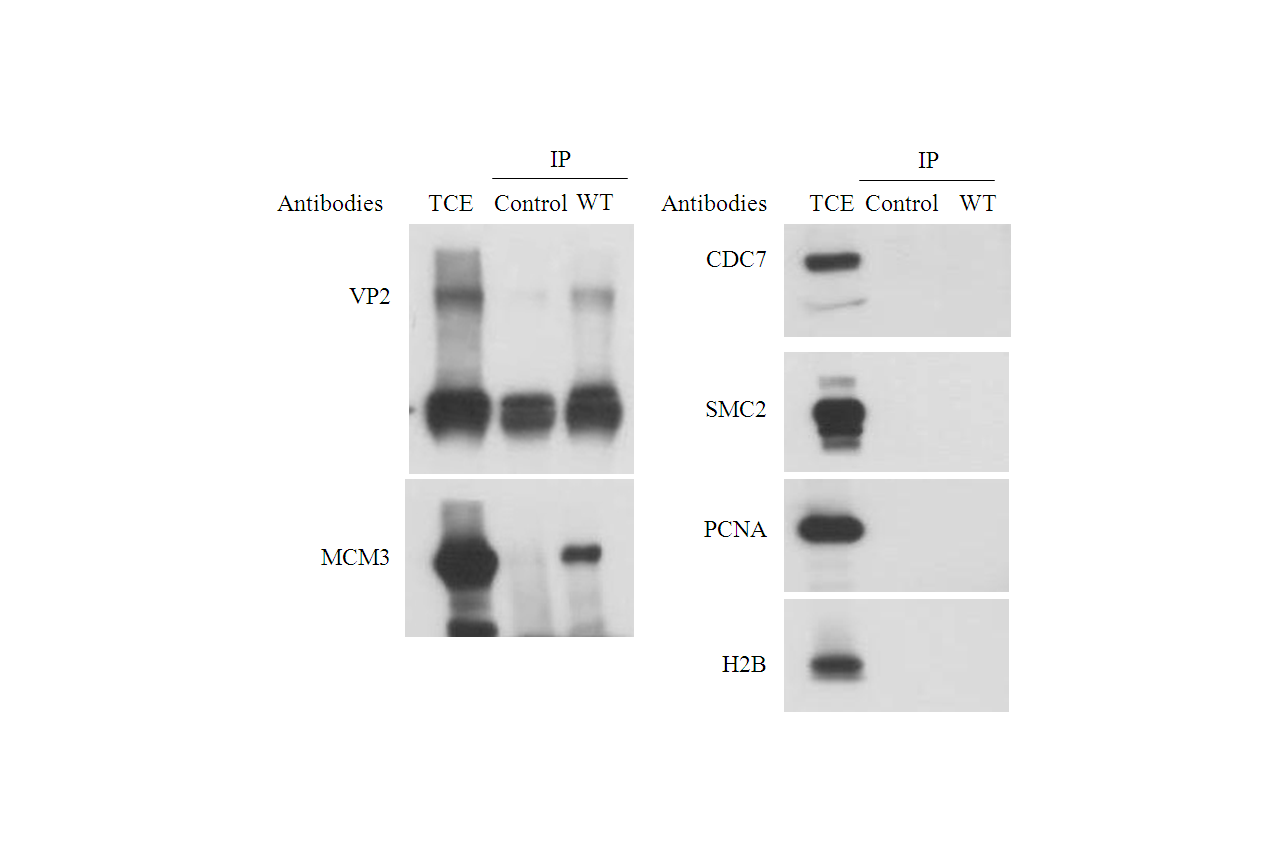

Supplement: Additional file 2 — Identification of protein-protein interactions with VP2 surveyed by co-immunoprecipitation. At 48 h post-transfection with plasmids encoding GFP (as the Control) or Flag-VP2-GFP (WT), cell lysates were immunoprecipitated by Flag M2 beads and immunoblotted against VP2, MCM3, CDC7, SMC2, PCNA, and H2B antibodies. The nuclear extracts containing VP2-GFP are designated as N. [file 1746-6148-8-15-S2.TIFF]
